# Morphological features of anterior segment: factors influencing intraocular pressure after cataract surgery in nanophthalmos

**DOI:** 10.1186/s40662-020-00212-4

**Published:** 2020-09-09

**Authors:** Qiang Lu, Wenwen He, Yi Lu, Xiangjia Zhu

**Affiliations:** 1 Department of Ophthalmology, Eye and Ear, Nose, and Throat Hospital, Fudan University, 83 Fenyang Road, Shanghai, 200031 People’s Republic of China; 2 Eye Institute, Eye and Ear, Nose, and Throat Hospital of Fudan University, Shanghai, 200031 People’s Republic of China; 3 Key Laboratory of Myopia, Ministry of Health, Shanghai, 200031 People’s Republic of China; 4 Shanghai Key Laboratory of Visual Impairment and Restoration, Shanghai, 200031 People’s Republic of China

**Keywords:** Nanophthalmos, Anterior segment, Intraocular pressure, Cataract surgery, Peripheral anterior synechiae, Boomerang-shaped iris, Iris crypt, Schlemm’s canal, Trabecular meshwork

## Abstract

**Purpose:**

To investigate the anterior segment in nanophthalmic eyes and their association with intraocular pressure after cataract surgery.

**Methods:**

Thirty-two nanophthalmic eyes (axial length [AL] < 18.5 mm) in 18 patients and 35 normal eyes (21 ≤ AL ≤ 24.5 mm) in 35 controls who had undergone uneventful cataract surgery were included. Swept-source optical coherence tomography was used to compare the anterior segment structures between the two groups. The associations between the anterior segment characteristics of nanophthalmic eyes and postoperative intraocular pressure (IOP) were also investigated.

**Results:**

The IOP-lowering effect of cataract surgery was remarkably insufficient in nanophthalmic eyes. Peripheral anterior synechiae (PAS) were observed in 56% (18/32) of nanophthalmic eyes, and a characteristic boomerang-shaped iris was observed in 28% (9/32). The anterior surface of the iris seemed “smoother” in nanophthalmic eyes than in normal eyes. Schlemm’s canal (SC) diameter, SC area, trabecular meshwork (TM) thickness, TM width, and TM area were generally smaller in the nanophthalmic eyes. Younger age, higher preoperative IOP, broader PAS, and smaller SC area were main contributors to higher postoperative IOP. AL and SC diameter may also be of great importance in IOP prediction in patients without glaucoma surgery and PAS.

**Conclusions:**

The morphological features of the anterior segment in nanophthalmic eyes are significantly different from those of normal eyes. Influencing factors such as age, AL, preoperative IOP, extent of PAS, SC and TM size could all be prognostic for IOP after cataract surgery in nanophthalmic eyes.

**Trial registration:**

ClinicalTrails.gov, Trial registration number: NCT02182921, Registered 8 July 2014.

## Background

Nanophthalmos is a developmental failure of the anterior neural tube or optic pit [1]. There was marked heterogeneity with respect to the definition of nanophthalmos; it was first defined by Duke-Elder [2] as an eye with two-thirds the normal volume and an axial length (AL) of 16.0–18.5 mm, and later studies used less strict definitions such as AL < 20.0 mm, < 20.5 mm and < 21.0 mm [3–6].

Due to the high lens/eye ratio, up to 54–77% of nanophthalmic eyes develop angle-closure glaucoma [5], which is the main cause of elevated intraocular pressure (IOP) in nanophthalmia. In eyes with angle-closure glaucoma, synechiae and a narrowing of the anterior chamber angle are direct and persistent causes of elevated IOP. Previous studies have demonstrated that broader peripheral anterior synechiae (PAS) can lead to a smaller reduction in IOP after phacoemulsification compared to that in eyes with fewer PAS [7]. The anterior positioning of the ciliary body and the dynamic physiological responses of the iris can also contribute to the narrowing of the chamber angle and possibly to PAS formation, as could be illustrated by medical imaging and iris crypts, respectively [8, 9].

Open-angle glaucoma in nanophthalmos also warrants investigation as even after the anterior chamber is deepened [5] and drainage is improved [10] by cataract surgery, the incidence of postoperative elevated IOP is still higher in these eyes than in normal eyes (33% [11] vs. 0.2% [12], respectively). Schlemm’s canal (SC) and the trabecular meshwork (TM) constitute the most important aqueous humor outflow pathway of the eyes, and pathological changes at these sites are closely associated with open-angle glaucoma. Despite these findings, it is still unclear why nanophthalmic eyes frequently suffer elevated IOP after cataract surgery.

Swept-source optical coherence tomography (SS-OCT) is a noninvasive technique that provides high-resolution images of the anterior chamber and the aqueous outflow pathway. It has shown excellent reproducibility and consistency for measuring anterior segment parameters [13]. Therefore, we used SS-OCT to investigate the morphological features of the anterior segment in nanophthalmic eyes using stricter criteria (≤ 18.5 mm) and their associations with IOP after cataract surgery.

## Materials and methods

### Ethics statement

The Institutional Review Board of the Eye and Ear, Nose, Throat (ENT) Hospital of Fudan University, Shanghai, China, approved this prospective study (NO.2013021). All procedures adhered to the Declaration of Helsinki and were conducted in accordance with the approved protocol. Clinical trial registration: NCT02182921 (www.clinicaltrials.gov).

### Patient selection

In this study, we define nanophthalmos as an eye with AL no more than 18.50 mm. Nanophthalmic patients (AL ≤ 18.50 mm) and age-matched controls (21.00 ≤ AL ≤ 24.50 mm) who had undergone uneventful cataract surgery at the Eye and ENT Hospital of Fudan University between January 1, 2016 and April 30, 2019 were included. The preoperative data and treatment records were obtained from the hospital records retrospectively. Patients underwent follow-up examinations 6 to 18 months after surgery. The exclusion criteria were eyes with no light perception, fixation failure, or a history of any other ocular surgery after the cataract surgery. Patients with pathological microcornea (corneal diameter < 10 mm) [14] or with other ocular or systemic abnormalities were also excluded from the study (as secondary ocular pathologies including high hyperopia, glaucoma or nystagmus could exist).

### Preoperative examinations

The preoperative ophthalmic examinations included the assessment of visual acuity, Goldmann applanation tonometry, corneal topography, B-scan ultrasonography, and the measurement of AL, anterior chamber depth (ACD), and lens thickness (LT) (IOLMaster 500, Carl Zeiss AG, Oberkochen, Germany). Corresponding results were retrospectively extracted from hospital records. The best-corrected visual acuity (BCVA) was recorded as a Snellen value and converted to the logarithm of the minimum angle of resolution (logMAR) for analysis. If visual acuity was counting fingers or worse, the corresponding conversion was calculated as follows: counting fingers = 2.0 logMAR, hand motion = 3.0 logMAR, and light perception was listed separately [15].

### Surgical technique

All procedures were performed by the same experienced surgeon (Prof. Yi Lu). A 2.2 mm temporal clear corneal incision was made after topical anesthesia. Viscoelastic (DisCoVisc; Alcon Laboratories, Inc., Fort Worth, TX, USA) was injected, followed by continuous curvilinear capsulorhexis, hydrodissection, and phacoemulsification, with folded intraocular lens (IOL) implanted. Goniosynechialysis was not performed during cataract surgery.

### Postoperative examinations

Routine ophthalmic examinations were performed during follow-up, including visual acuity, Goldmann applanation tonometry, slit-lamp examination and fundoscopy. An IOP change greater than 0.5 mmHg was regarded as valid after the calibration error was taken into consideration [16]. Therefore, when the follow-up IOP was compared with the preoperative IOP, a decline of more than 0.5 mmHg was deemed a reduction in IOP.

The SS-OCT (CASIA SS-1000; Tomey Corporation, Nagoya, Japan) examinations of the anterior segment were performed in the dark without mydriasis. The three-dimensional (3D) anterior segment mode was used for whole-range scanning in the PAS analysis. Another 3D angle high-definition mode was performed separately in each of the four quadrants (nasal, temporal, superior, and inferior) for further angle analysis [17]. The patients were told to sit and stare at one of four fixation lights in turn during the test.

**Fig. 1:**
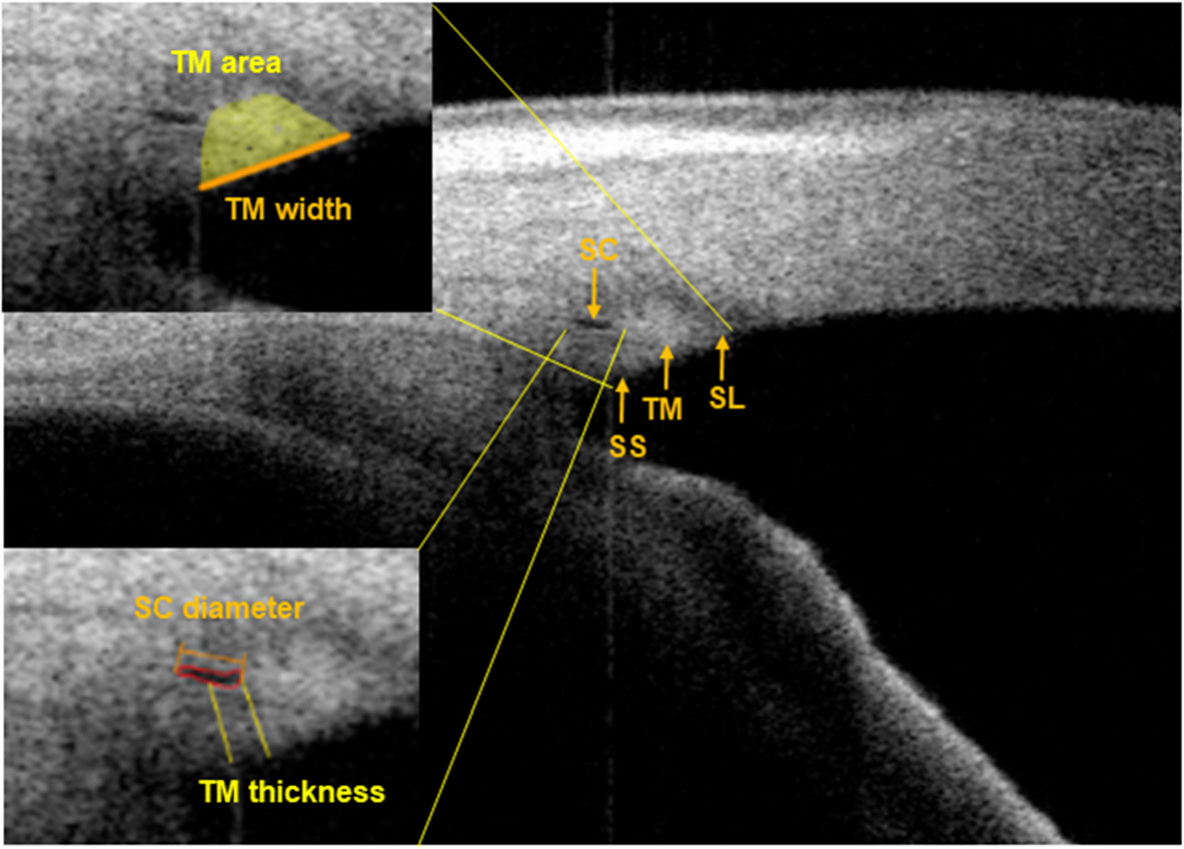
Measurement of Schlemm’s canal (SC) and the trabecular meshwork (TM). The boundary of SC is drawn freehand (red outline) in the enlarged view in the lower left corner. The SC diameter is measured from the posterior to the anterior SC (orange line) and the SC area is the black oval space surrounded by the red line. The TM thickness is measured at the anterior end point and halfway down SC (yellow line). The enlarged view in the upper left corner shows the TM area (yellow) and TM width (orange line). The TM width is the length between the scleral spur (SS) and Schwalbe’s line (SL)

We evaluated the following morphological features of the anterior segment: synechiae and the narrowing of the anterior chamber angle, the characteristics of the iris, and the changes in SC and TM. The extent of PAS was measured using the 3D view function in SS-OCT and recorded by degree of angle [7]. The morphology of the iris was described, and a “boomerang-shaped” iris was defined as a “bended” iris shown in cross-section which is different from a normal straight phenotype. The iris crypts were graded in each eye using a slit lamp and a previously described grading system was used: grade 1 (no crypts); grade 2 (1–3 crypts); grade 3 (≥ 4 crypts, < 1 mm in diameter); grade 4 (≥ 4 crypts, ≥ 1 mm in diameter), and grade 5 (numerous crypts, > 1 mm in diameter, covering nearly the entire iris) [8]. The diameter and area of the SC, and the thickness, width, and area of the TM were measured manually by two independent observers (QL, WH) with the Image Processing and Analysis in Java (ImageJ) software (www.imagej.nih.gov/ij/; National Institutes of Health, Bethesda, MD, USA). Both observers were masked to the identities of the subject. The percentage of observable SC was calculated as the number of eyes with observable SC/total number of eyes observed × 100%. SC was defined as a thin, black, lucent space. The SC diameter was defined as the meridional length, which is the distance from the posterior to the anterior SC. The SC area was defined as the area of the black oval space. The TM was seen as a hyperreflective area surrounded by a hyporeflective arc [18]. The thickness of the TM was calculated as the average of two measurements made at the anterior end point and halfway down SC, as previously reported [17, 19]. The width of the TM was defined as the distance between the scleral spur and Schwalbe’s line, and the TM area as the hyperreflective area [17] (Fig. 1).

### Statistical analysis

Statistical analyses were performed with SPSS version 19.0 (SPSS Inc., Chicago, IL, USA) and SAS version 9.4 (SAS Institute, Cary, NC, USA). Quantitative data are presented as means ± standard deviations (SD). The χ^2^ test was used to evaluate categorical variables. The reproducibility of SC and TM measurements was assessed by the intraclass correlation coefficient (ICC) whose difference between groups was tested with R 3.1.0 using the *cocor* package. The generalized estimating equation (GEE) approach is an extension of linear regression and is useful in ophthalmic studies to compare measurements made in the two eyes of the same person. It was used to evaluate the differences between nanophthalmic and normal eyes. A series of GEE models were also used in testing the univariate associations between postoperative IOP and all covariates. All variables with a univariate *P* value < 0.20 were considered in the multivariate GEE regression models, with backwards selection used to determine the final model. A paired *t* test was used to evaluate the paired ocular parameters before and after the surgery. *P* values of less than 0.05 were considered statistically significant.

## Results

### Baseline characteristics

Thirty-eight nanophthalmic eyes of 20 patients and 35 normal eyes of 35 age-matched controls were included in the follow-up. Four of the 38 nanophthalmic eyes had severe postoperative complications, including 2 malignant glaucoma, 1 exudative ciliochoroidal detachment and 1 IOL subluxation, and all ended up with no light perception by the time of follow-up. Two other nanophthalmic eyes also lost light perception without specific complications. Therefore, these eyes without light perception were excluded from the final data analysis due to fixation failure i.e., 32 nanophthalmic eyes of 18 patients were used. Among which, 5 eyes (16%) of 3 nanophthalmic patients were treated with IOP-lowering medications at follow-up. For these eyes, the follow-up IOPs were recorded as the premedication IOPs obtained from their postoperative medical records.

Demographic data for the two groups are shown in Table 1. There were no statistically significant differences between the two groups in terms of age, sex, eye laterality, central corneal thickness (CCT), LT or follow-up time. Both AL and ACD were significantly shorter in nanophthalmic eyes (GEE, *P* < 0.001). Preoperative BCVA were significantly worse in nanophthalmic eyes than in normal eyes (GEE, *P* < 0.001). No difference was observed in preoperative IOP between the two groups; 17 nanophthalmic eyes (53%) had undergone glaucoma surgery before the cataract surgery, among which 14 of them had laser peripheral iridotomy (LPI) and 3 of them had trabeculectomy.

**Table 1:** Baseline characteristics of patients and eyes^a^

	Nanophthalmic eyes (*N* = 32)	Normal eyes (*N* = 35)	*P* value
Age (years)	52.83 ± 20.29	57.91 ± 12.79	0.320
Sex (male/female)	6/12	12/23	1.000
Eye (OD/OS)	15/17	17/18	0.890
CCT (μm)	550.16 ± 48.85 (478.00–664.00)	542.51 ± 26.92 (504.00–602.00)	0.642
AL (mm)	16.87 ± 1.02 (15.32–18.49)	23.09 ± 0.88 (21.67–24.50)	< 0.001^b^
ACD (mm)	1.75 ± 0.50 (0.97–3.10)	2.88 ± 0.51 (2.12–3.96)	< 0.001^b^
LT (mm)	4.70 ± 0.37 (3.98–5.63)	4.64 ± 0.40 (4.00–5.53)	0.574
Preoperative BCVA (logMAR)	1.21 ± 0.77, 1 light perception	0.52 ± 0.16	< 0.001^b^
Follow-up BCVA (logMAR)	1.03 ± 0.74, 1 light perception	0.13 ± 0.60	< 0.001^b^
Preoperative IOP (mmHg)	16.13 ± 3.72 (11.0–24.0)	15.62 ± 2.59 (8.9–19.6)	0.612
Follow-up period (month)	13.4 ± 3.3	12.2 ± 3.8	0.133

*CCT* =  central corneal thickness, *AL* =  axial length, *ACD* =  anterior chamber depth, *LT* =  lens thickness, *BCVA* =  best-corrected visual acuity, *logMAR* =  logarithm of the minimum angle of resolution, *IOP* =  intraocular pressure

^a^ Data are presented as means ± standard deviations, and the ranges are listed below. Generalized estimating equations were used to test the differences in age, CCT, AL, BCVA, IOP and follow-up time between nanophthalmic eyes and normal eyes. The χ2 test was used to test the differences in the distributions of sex and eye laterality

^b^ Statistically significant (*P* < 0.05)

### IOP and visual outcomes

**Fig. 2:**
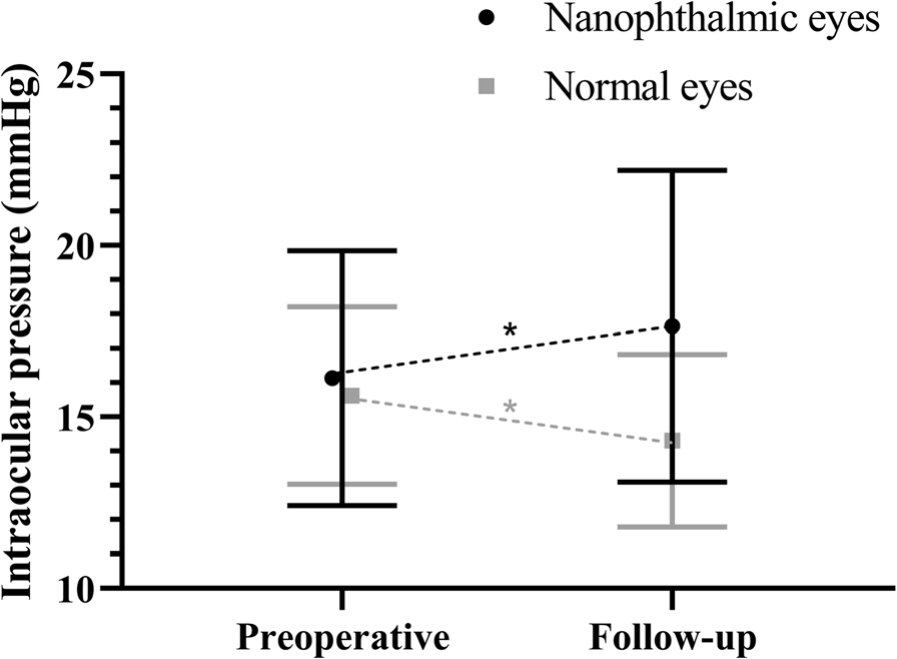
Intraocular pressure change in nanophthalmic eyes and normal eyes after cataract surgery. *P < 0.05

Postoperatively, BCVA in nanophthalmic eyes remains worse compared with normal eyes (1.03 ± 0.74 logMAR in nanophthalmic eyes and 0.13 ± 0.60 logMAR in normal eyes; GEE, *P* < 0.001). There was a significant improvement in BCVA in the normal eyes (paired *t* test, *P* < 0.001), but not in the nanophthalmic eyes (paired *t* test, *P* = 0.051). As for the IOP after cataract surgery, higher IOP was observed in the nanophthalmic group (17.65 ± 4.55 mmHg in nanophthalmic eyes and 14.30 ± 2.52 mmHg in normal eyes; GEE, *P* = 0.001). A reduction in IOP could be seen in normal eyes (− 1.32 ± 2.23; paired *t* test, *P* = 0.001), whereas there was a slight elevation in IOP in the nanophthalmic eyes (1.52 ± 3.43; paired *t* test, *P* = 0.017; Fig. 2). A reduction in IOP was observed in 74% (26/35) of normal eyes compared with just 25% (8/32) of nanophthalmic eyes. An increase in IOP was observed in 17% (6/35) of normal eyes while 63% (20/32) of nanophthalmic eyes had an IOP elevation after cataract surgery (χ^2^ test, *P* < 0.001). In those nanophthalmic eyes with increased IOP after cataract surgery (63% [20/32]), broader PAS, smaller SC area, thinner TM and smaller TM area could be observed (GEE, *P* = 0.001, 0.004, 0.012 and 0.018 respectively). No differences were seen in normal eyes among groups with distinguished IOP change.

### PAS, narrowing of anterior chamber angle, and iris morphology

**Fig. 3:**
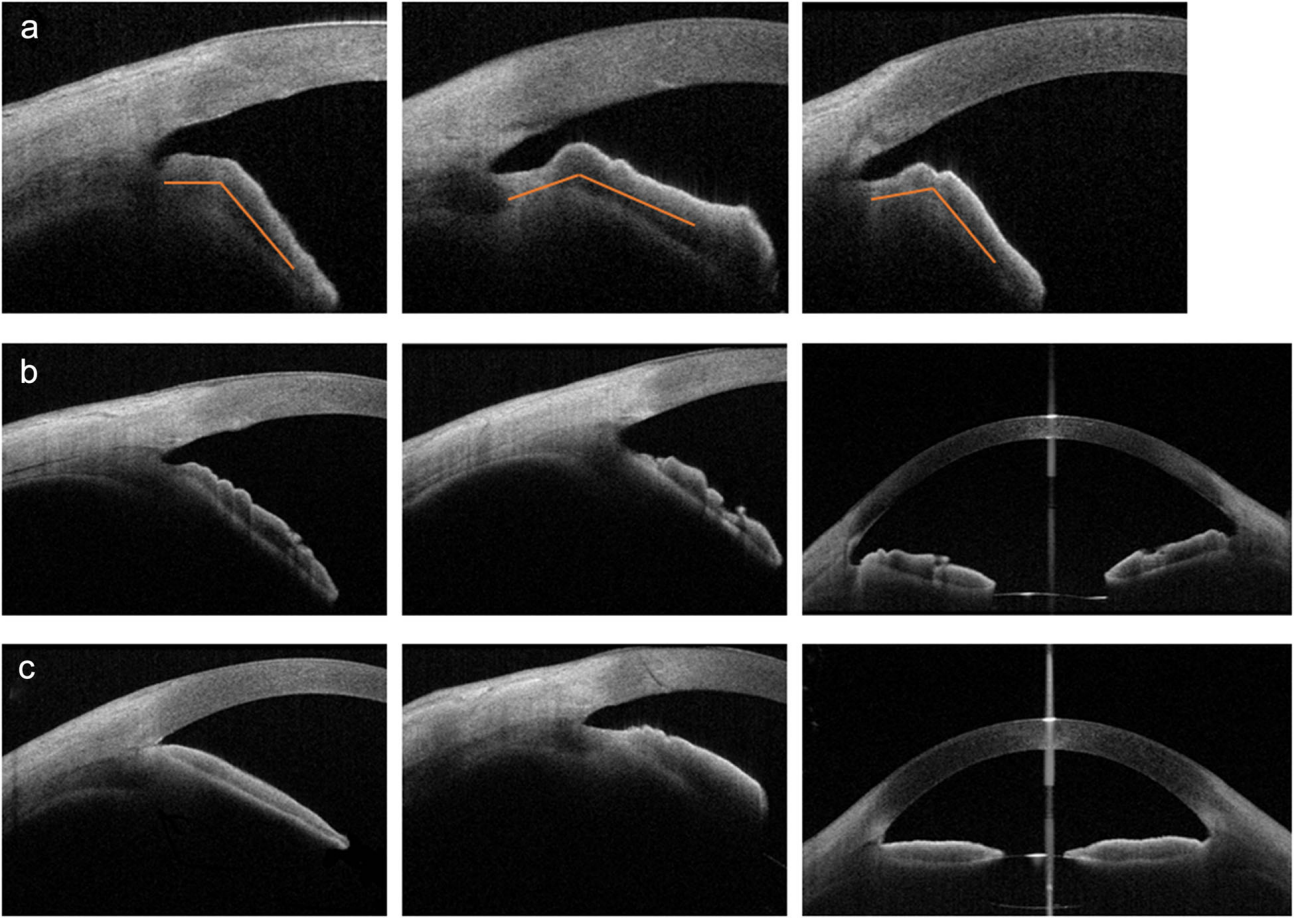
Different iris morphologies in nanophthalmic eyes and normal eyes. **a** Representative boomerang-shaped iris from three different nanophthalmic patients. Orange lines indicate the irregular angle formed by the boomerang-shaped iris. **b** Representative images of irises from three different controls. **c** Representative images of irises from three different nanophthalmic patients

Different extents of PAS were observed in 56% (18/32) of the nanophthalmic eyes, with 19% (6/32) having a PAS ≤ 90°, 6% (2/32) having a PAS between 90° and 180°, 9% (3/32) having a PAS between 180° and 270°, and 22% (7/32) having a PAS > 270°. No PAS was observed in the control group. In addition to direct iridotrabecular contact, the narrowing of the anterior chamber angle caused by the characteristic boomerang-shaped iris was observed in 28% (9/32) of the nanophthalmic eyes (Fig. 3a), whereas this phenomenon was not observed in normal eyes. The follow-up IOP of the nanophthalmic eyes did not differ between eyes with and without this phenotype (GEE, *P* = 0.790). The iris tended to be “smoother” on the anterior surface in the nanophthalmic eyes than in the normal eyes (Fig. 3b–c). The grading of the iris crypts showed no difference between the two groups (χ^2^ test, *P* = 0.447; Table 2).

**Table 2:** Comparison of iris features between nanophthalmic and normal eyes^a^

	Nanophthalmic eyes (*N* = 32)	Normal eyes (*N* = 35)	*P* value
Extent of PAS			
0° (no PAS)	44% (14/32)	100% (35/35)	< 0.001^b^
0° to ≤90°	19% (6/32)	0	
> 90° to ≤180°	6% (2/32)	0	
> 180° to ≤270°	9% (3/32)	0	
> 270° to ≤360°	22% (7/32)	0	
Grading of iris crypts			
1	63% (20/32)	49% (17/35)	0.447
2	28% (9/32)	43% (15/35)	
3	9% (3/32)	9% (3/35)	
4	0%	0%	
5	0%	0%	

^a^ Data are presented as percentages (proportions). χ^2^ test was used to test the distribution of the extent of peripheral anterior synechiae (PAS) and the grading of visible crypts

^b^ Statistically significant (*P* < 0.05)

### SC and TM parameters

**Fig. 4:**
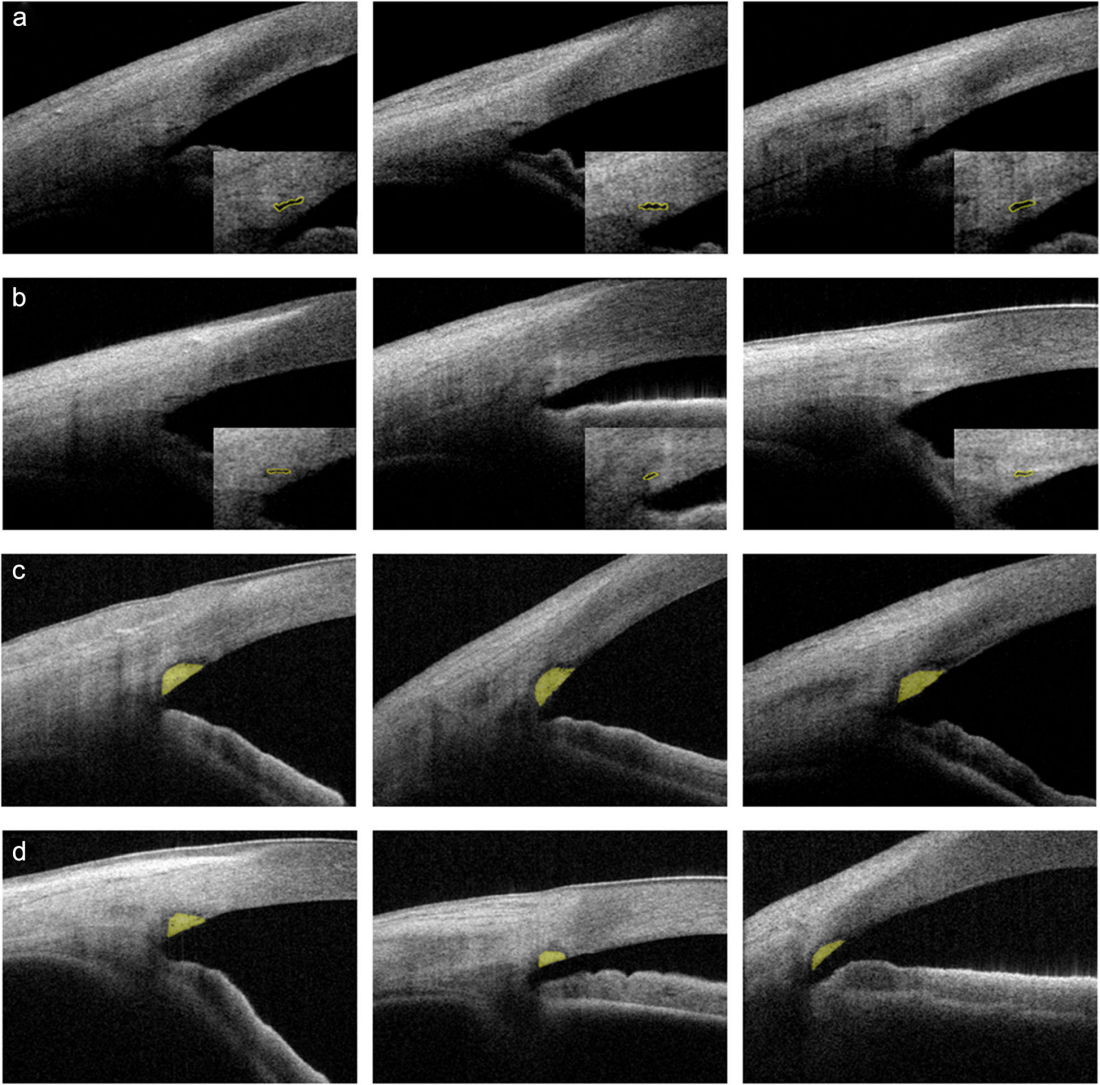
The Schlemm’s canal (SC) and trabecular meshwork (TM) are smaller in nanophthalmic eyes compared with normal eyes. **a** Representative images of SC (yellow outline) from three different control patients. **b** Representative images of SC from three different nanophthalmic patients. **c** Representative images of the TM (yellow area) from three different controls. **d** Representative images of the TM from three different nanophthalmic patients. All images were taken in the nasal quadrant

As shown in Table 3, the SC was generally less visible in the nanophthalmic eyes than in the normal eyes. For inter-observer repeatability, the ICC values were 0.834, 0.952, 0.834, 0.824 and 0.738 for SC diameter, SC area, TM thickness, TM width and TM area, respectively. No difference was observed between groups with regards to ICC (Table 3). Both the SC diameter and the SC area were significantly smaller in all four quadrants of the nanophthalmic eyes than in the controls (Table 3 and Fig. 4a–b). The mean TM thickness, width, and area of the four quadrants combined were significantly smaller in the nanophthalmic eyes than in the normal eyes. When the different quadrants were considered individually, the TM thickness and width were significantly smaller in the nasal quadrant of the nanophthalmic eyes than in that of the controls. The TM area was significantly smaller in the nanophthalmic eyes than in the normal eyes in almost all quadrants, except for the temporal quadrant (Table 3 and Fig. 4c–d).

**Table 3:** Parameters of Schlemm’s canal and trabecular meshwork in the four quadrants of nanophthalmic and normal eyes

		Visibility (%)^a^	SC diameter (μm)^b^	SC area (μm^2^)^b^	TM thickness (μm)^b^	TM width (μm)^b^	TM area (μm^2^)^b^
Nasal	Nano	87.5	136.18 ± 29.58	2807.18 ± 945.41	99.20 ± 23.54	502.43 ± 65.75	38,065.32 ± 9882.56
	Cont	100	165.11 ± 26.97	4264.03 ± 784.64	133.54 ± 25.11	542.66 ± 45.08	46,887.89 ± 6124.66
	*P*	0.031^c^	< 0.001^c^	< 0.001^c^	< 0.001^c^	0.006^c^	< 0.001^c^
Temporal	Nano	93.8	147.77 ± 30.19	3086.23 ± 729.26	115.87 ± 33.72	508.03 ± 66.92	41,779.33 ± 10,536.28
	Cont	100	173.23 ± 29.14	4417.20 ± 776.42	129.99 ± 26.66	531.23 ± 40.73	45,837.11 ± 6163.98
	*P*	0.133	0.001^c^	< 0.001^c^	0.066	0.129	0.078
Superior	Nano	81.3	142.15 ± 23.95	3095.31 ± 689.14	121.44 ± 34.91	515.38 ± 66.69	41,776.92 ± 6991.90
	Cont	100	171.51 ± 25.04	4422.46 ± 888.13	124.54 ± 24.66	547.2 ± 37.40	45,282.17 ± 5921.97
	*P*	0.007^c^	< 0.001^c^	< 0.001^c^	0.573	0.065	0.067
Inferior	Nano	84.4	140.63 ± 31.65	3218.56 ± 901.31	126.46 ± 22.09	516.44 ± 58.51	40,955.81 ± 6291.93
	Cont	97.1	172.35 ± 22.91	4545.38 ± 802.46	133.09 ± 20.74	539.91 ± 34.76	48,541.62 ± 4737.57
	*P*	0.068	< 0.001^c^	< 0.001^c^	0.189	0.058	< 0.001^c^
Average	Nano	86.7	141.16 ± 23.05	3041.94 ± 622.59	115.35 ± 21.77	511.08 ± 46.14	40,701.00 ± 6762.56
	Cont	99.3	170.34 ± 15.57	4403.73 ± 616.48	130.45 ± 16.26	538.75 ± 23.62	46,611.91 ± 4075.82
	*P*	0.047^c^	< 0.001^c^	< 0.001^c^	0.002^c^	0.006^c^	< 0.001^c^
ICC	Nano	NA	0.81	0.96	0.83	0.82	0.72
	Cont	NA	0.77	0.89	0.80	0.77	0.62
	*P*	NA	0.38	0.14	0.49	0.46	0.44

*Nano* = nanophthalmic group (*N* = 32), *Cont* =  control group (*N* = 35), *P =  P* value, *ICC* intraclass correlation coefficient, *NA* =  not applicable, *SC*= Schlemm’s canal, *TM*= trabecular meshwork

^a^ Comparisons of nanophthalmic group and control group were made using the χ^2^ test

^b^ Comparisons of the nanophthalmic and control groups were made with generalized estimating eq. ICC-related parameters were tested with R 3.1.0 using the *cocor* package

^c^ Statistically significant (*P* < 0.05)

### Prognostic factors for postoperative IOP in nanophthalmic eyes

The correlations between the 17 factors tested (age, sex, eye laterality, CCT, AL, ACD, LT, preoperative IOP, history of glaucoma surgery (0 = without glaucoma surgery; 1 = LPI; 2 = trabeculectomy), extent of PAS, boomerang-shaped iris (0 = without; 1 = with), iris crypt grading, SC diameter, SC area, TM thickness, TM width, and TM area) and follow-up IOP in nanophthalmic eyes were analyzed with univariate GEE analyses. Variables significantly associated with postoperative IOP included preoperative IOP, history of glaucoma surgery, the extent of PAS, SC area, and TM area (*P* < 0.001, = 0.001, < 0.001, = 0.004 and = 0.032 respectively, Table 4). Variables considered in the multivariate GEE model of postoperative IOP included age, CCT, preoperative IOP, history of glaucoma surgery, extent of PAS, SC area, TM thickness, and TM area. The final model has an R^2^ value of 0.82 (*F* = 22.55, *P* < 0.001), and consisted age, preoperative IOP, extent of PAS, SC area as well as TM thickness (Table 4). Notably, in addition to the prognostic factors mentioned above, AL though not significant in IOP prediction in the overall nanophthalmic eyes, served as an essential prognostic factor for postoperative IOP in nanophthalmic eyes without any history of glaucoma surgery and without PAS in multivariate GEE regression models (Supplementary Tables 1 and 2). Smaller SC diameter could also contribute to higher IOP postoperative in both subgroups of nanophthalmic eyes (Supplementary Tables 1 and 2).

**Table 4:** Estimated mean differences in postoperative IOP in nanophthalmos based on GEE models for all variables

Variable	Univariate GEE models		Multivariate GEE models	
	Beta ^a^	*P* value	Beta ^a^	*P* value
Age	−0.076 ± 0.040	0.056	− 0.069 ± 0.018	< 0.001^b^
Gender	NA	0.391		
Eye laterality	NA	0.868		
CCT	0.021 ± 0.016	0.185		
AL	−0.526 ± 0.866	0.544		
ACD	0.256 ± 1.086	0.814		
LT	2.168 ± 2.329	0.352		
Preoperative IOP	0.824 ± 0.175	< 0.001^b^	0.662 ± 0.102	< 0.001^b^
Glaucoma surgery	NA	0.001^b^		
Extent of PAS	0.018 ± 0.004	< 0.001^b^	0.012 ± 0.004	0.003^b^
Boomerang-shaped iris	NA	0.790		
Iris crypt grading	0.857 ± 1.938	0.658		
SC diameter	−0.032 ± 0.041	0.437		
SC area	−0.003 ± 0.001	0.004^b^	−0.002 ± 0.001	0.001^b^
TM thickness	−0.043 ± 0.030	0.149	0.040 ± 0.023	0.090
TM width	−0.012 ± 0.019	0.524		
TM area	0.0003 ± 0.0001	0.032^b^		

*IOP* =  intraocular pressure, *GEE* =  generalized estimating equation, *NA* =  not applicable, *CCT* =  central corneal thickness, *AL* =  axial length, *ACD* =  anterior chamber depth, *LT* =  lens thickness, *PAS* =  peripheral anterior synechiae, *SC* =  Schlemm’s canal, *TM* =  trabecular meshwork

^a^ Data represent the mean changes ± standard error in postoperative IOP anticipated for each factor. GEE analysis was used

^b^ Statistically significant (*P* < 0.05)

## Discussion

Nanophthalmos is a type of ocular dysgenesis that is usually accompanied by structural abnormalities in the anterior segment [20]. Previous studies of nanophthalmos have focused on its classification and related complications [5, 11]. However, very few studies have investigated the anatomical anomalies in the anterior segment of these eyes and their associations with IOP after cataract surgery. In this study, we included patients with nanophthalmic eyes that met Duke-Elder’s criteria [2] and demonstrated that the IOP-lowering effect of cataract surgery was insignificant in these eyes. SS-OCT revealed broader PAS, iris structural abnormalities, and less-well-developed SC and TM in the nanophthalmic eyes than in the normal eyes. The multivariate analysis also showed that younger age, shorter AL, higher preoperative IOP, broader PAS, and smaller SC were significantly associated with higher IOP in nanophthalmic eyes after cataract surgery.

The incidence of complications and complete visual loss is more frequent in the nanophthalmic group. Malignant glaucoma is one of the most serious complications of cataract surgery in patients with angle closure, and has been reported to occur in 6.8% of cases in microphthalmic eyes [21]. Here, 2/38 eyes (5.3%) from the same patient were diagnosed with malignant glaucoma and ended up in visual loss. Zonular abnormality may exist in this patient and could not provide sufficient support for IOL, together with the pressure from the vitreous, the IOL was pushed forward [11]. The zonular abnormality in nanophthalmos may also contribute to IOL subluxation. As for the exudative retinal detachment, it was well documented in nanophthalmos and has an incidence rate of 2.9% [21]. In this study, one of 38 eyes (2.6%) experienced uveal effusion and lost light perception in the long run. Of note, these six patients who ended up with light deprivation generally had bad vision or only had suspicious light perception preoperatively, and a postoperative gradual loss of vision did not warrant going to the hospital.

The IOP-lowering effect of cataract surgery in nanophthalmic eyes was insufficient compared with that achieved in normal eyes. In fact, a slight increase in postoperative IOP was observed in the nanophthalmic eyes. Phacoemulsification has long been regarded as an effective approach to IOP reduction in patients with either angle-closure glaucoma or open-angle glaucoma [7, 22], but is generally unsuccessful in nanophthalmic eyes. The morphological anomalies of the anterior segment in these cases may be important in terms of changes in postoperative IOP.

Although cataract surgery effectively relieves the crowding of the anterior chamber [23], it is not useful in dealing with chronic PAS [24]. PAS are considered to be a direct and persistent cause of elevated IOP, especially in eyes with chronic angle-closure glaucoma [25]. Even with goniosynechialysis, the outcome can be unsatisfying for chronic PAS because the success rate of goniosynechialysis is low, these eyes are more susceptible to recurrent PAS, and the creeping adhesion of the iris tissue into the intertrabecular space may cause irreversible damage to the TM [7, 24]. Therefore, any untreated PAS could contribute to the unfavorable postoperative IOP in nanophthalmic eyes. Further studies are needed to evaluate the efficacy of goniosynechialysis in nanophthalmic eyes.

Iridal abnormalities may also contribute to the unsatisfactory postoperative IOP status of nanophthalmic eyes. The characteristic boomerang-shaped iris in nanophthalmic eyes is thought to be an indirect indicator of anteriorly positioned ciliary processes. This physical displacement of the peripheral iris is associated with angle-narrowing and could result in goniosynechiae [26]. Hypertrophy of the ciliary body resulting from postoperative inflammation [25], annular ciliochoroidal effusion, or ciliary body detachment [27], and the relatively small space in the anterior segment in nanophthalmic eyes could result in the anterior positioning of the ciliary processes. However, in the present study, this characteristic iris shape was not significantly correlated with the follow-up IOP in nanophthalmic eyes. This may be attributable to the limited sample size and the low diagnostic rate of anteriorly positioned ciliary processes with SS-OCT compared with the traditionally used ultrasound biomicroscopy [28].

Iris crypts play a vital role in aqueous humor outflow by affecting the permeability of the anterior iris surface, the iris volume and curvature, and the uveoscleral and trabecular pathways [8]. Previous studies have shown that fewer iris crypts is a surrogate marker for a static biometric risk factor for angle closure [8]. According to our observations, the anterior surface of the iris was generally “smoother” and more “rigid” in small eyes when seen in cross-section, but in this study, the grades of the iris crypts did not differ between the two groups. The iris crypt grading was also independent of IOP. Previous studies of iris crypts have only revealed a possible correlation with the progression of angle-closure glaucoma, but no direct relationship with IOP was found [8], suggesting that crypts are not as important as the trabecular pathway in the aqueous humor outflow. However, when the “smooth” and “rigid” anterior surface of the iris on SS-OCT images and the intrinsic dysgenesis of the anterior segment in nanophthalmic eyes are considered, we believe that this characteristic of the iris warrants further study and that better quantitative criteria could be applied.

The trabecular pathway, involving SC and TM, is the primary drainage route of the aqueous humor and is intimately related to the pathophysiology of glaucoma [19]. The SC diameter and area were significantly smaller in all four quadrants in nanophthalmic eyes, and the SC area was negatively correlated with postoperative IOP. We speculate that the intrinsic dysgenesis of the outflow pathway in nanophthalmic eyes could contribute to their elevated IOP, and that the developmental-anomaly-related failure of ciliary muscle movement may diminish the traction on SC [1, 29]. These resistance-increasing factors could lead to the elevation of IOP, which may cause SC to collapse and the TM to compress, thus initiating a vicious cycle of progressively increasing IOP [19]. In terms of the TM parameters, previous studies have demonstrated that the aqueous outflow is segmental and that the TM is thicker in regions of active flow [17]. In the present study, the TM parameters were generally smaller in the nanophthalmic eyes than in the normal eyes. The diminution of the TM in nanophthalmic eyes may be explained by its dysgenesis, the metabolic dysfunction of the intraocular fluid after surgery, and elevated IOP [30–32]. The relatively thinner TM in nanophthalmic eyes may indicate inactive aqueous outflow, which could explain the elevated IOP after cataract surgery. However, further studies with larger samples are required to confirm this possibility.

Our multivariate regression analysis showed that younger age, higher preoperative IOP, broader PAS, and smaller SC area were independently associated with elevated postoperative IOP in nanophthalmic eyes after cataract surgery. The significant negative contribution of age to the follow-up IOP may be attributable to the reduced production of aqueous humor with age, as supported by the majority of related studies in Asia [33], whereas contrasting results have been obtained in non-Asian studies [34]. Furthermore, preoperative IOP has long been regarded as a reliable predictor of postoperative IOP in previous studies [35].

Controversy remains regarding the relationship between ACD and IOP change after cataract surgery. Some studies claimed that preoperative ACD did not have a significant relationship with postoperative IOP, while some others argued that shallower ACD was associated with a more pronounced IOP reduction [36]. However, there were also studies which suggested that shallower ACD could point to underdeveloped anterior segment and TM, and thus leading to higher postoperative IOP [37]. In this study, ACD did not play an important role in the prognosis of postoperative IOP in the whole nanophthalmic population but is negatively correlated with postoperative IOP in nanophthalmic patients with no history of glaucoma surgery. We believe that after ruling out the interference of glaucoma surgery, pre-existing shallow ACD together with smaller AL indicated the disproportion of small eyes as well as dysgenesis of the anterior segment, which increases the risk for higher postoperative IOP in nanophthalmic eyes.

Nanophthalmos is usually accompanied by various structural abnormalities. The insignificant BCVA improvement in nanophthalmic eyes and its significant lower BCVA compared with normal eyes might have been owing to amblyopia, foveal hypoplasia [38], pigmentation retinopathy [39] or the small sample size. Controversy remains as to whether cataract surgery could bring improvement to BCVA in nanophthalmic patients [11, 40], and further studies should be carried out to confirm the results.

## Conclusions

In conclusion, the morphological features of the anterior segment could be important determinants of the insufficient IOP-lowering effect of cataract surgery in nanophthalmic eyes. Nanophthalmic eyes tend to have broader PAS, structural abnormalities of the iris, and less well-developed SC and TM. Younger age, higher preoperative IOP, broader PAS and smaller SC area were main prognostic factors for higher IOP after cataract surgery in nanophthalmic eyes. AL and SC diameter could also be predictive to IOP outcomes in patients without history of glaucoma surgery and PAS.

## Supplementary information

**Additional file 1: Supplementary Table 1.** Estimated mean differences in postoperative IOP in nanophthalmic eyes without previous glaucoma surgery based on GEE models for all variables.

**Additional file 2: Supplementary Table 2.** Estimated mean differences in postoperative IOP in nanophthalmic eyes without peripheral anterior synechiae based on GEE models for all variables.

## Data Availability

The datasets generated and/or analyzed during the present study are not publicly available (obtained from Eye and Ear, Nose, and Throat Hospital, Fudan University, Shanghai repository), but are available from the corresponding author upon reasonable request.

## Abbreviations

IOP: Intraocular pressure
AL: Axial length
PAS: Peripheral anterior synechiae
SC: Schlemm’s canal
TM: Trabecular meshwork
SS-OCT: Swept-source optical coherence tomography
ENT: Ear, Nose and Throat
IOL: Intraocular lens
BCVA: Best-corrected visual acuity
LogMAR: Logarithm of the minimum angle of resolution
3D: Three-dimensional
ImageJ: Image Processing and Analysis in Java
SD: Standard deviations
ICC: Intraclass correlation coefficient
GEE: Generalized estimating equation
CCT: Central corneal thickness
LPI: Laser peripheral iridotomy
