# Supplementary material for: Morphological features of anterior segment: factors influencing intraocular pressure after cataract surgery in nanophthalmos
Source: Eye Vis (Lond). 2020 Sep 9;7:47. doi: 10.1186/s40662-020-00212-4 (PMC7495875; doi:10.1186/s40662-020-00212-4)
Supplement: Supplementary file 1 — Additional file 1: Supplementary Table 1. Estimated mean differences in postoperative IOP in nanophthalmic eyes without previous glaucoma surgery based on GEE models for all variables. [file 40662_2020_212_MOESM1_ESM.docx]

| **Supplementary Table 1. Estimated mean differences in postoperative IOP in nanophthalmic eyes**  **without previous glaucoma surgery based on GEE models for all variables.** | | | | |
| --- | --- | --- | --- | --- |
| **Variable** | **Univariate GEE models** | | **Multivariate GEE models** | |
|  | **Beta** ^a^ | ***P* value** | **Beta** ^a^ | ***P* value** |
| Age | -0.112 ± 0.045 | 0.012^b^ | -0.244 ± 0.002 | <0.001^b^ |
| Gender | NA | 0.141 | 3.038 ± 0.171 | <0.001^b^ |
| Eye laterality | NA | 0.122 |  |  |
| CCT | 0.045 ± 0.013 | <0.001^b^ | 0.011 ± 0.002 | <0.001^b^ |
| AL | -5.780 ± 0.142 | <0.001^b^ | -0.578 ± 0.058 | <0.001^b^ |
| ACD | -7.182 ±0.143 | <0.001^b^ | -6.157 ±0.270 | <0.001^b^ |
| LT | -0.955 ± 1.149 | 0.406 |  |  |
| Preoperative IOP | 0.682 ± 0.418 | 0.103 | 0.711 ± 0.025 | <0.001^b^ |
| Extent of PAS | 0.020 ± 0.007 | 0.002^b^ |  |  |
| Boomerang-shaped iris | NA | 0.127 |  |  |
| Iris crypt grading | -0.627 ± 1.023 | 0.540 |  |  |
| SC diameter | -0.122 ± 0.047 | 0.010^b^ | -0.041 ± 0.006 | <0.001^b^ |
| SC area | -0.004 ± 0.001 | <0.001^b^ | 0.001 ± 0.0002 | <0.001^b^ |
| TM thickness | -0.017 ± 0.015 | 0.149 |  |  |
| TM width | -0.022 ± 0.021 | 0.524 |  |  |
| TM area | -0.0003 ± 0.0002 | 0.101^b^ |  |  |
| IOP = intraocular pressure; GEE = generalized estimating equation; NA = not applicable; CCT = central corneal thickness; AL= axial length; ACD = anterior chamber depth; LT = lens thickness; PAS = peripheral anterior synechiae; SC = Schlemm's canal; TM = trabecular meshwork.  ^a^ Data represent the mean changes ± standard error in postoperative IOP anticipated for each factor. GEE analysis was used.  ^b^ Statistically significant (*P* < 0.05). | | | | |
