# Supplementary material for: Morphological features of anterior segment: factors influencing intraocular pressure after cataract surgery in nanophthalmos
Source: Eye Vis (Lond). 2020 Sep 9;7:47. doi: 10.1186/s40662-020-00212-4 (PMC7495875; doi:10.1186/s40662-020-00212-4)
Supplement: Supplementary file 2 — Additional file 2: Supplementary Table 2. Estimated mean differences in postoperative IOP in nanophthalmic eyes without peripheral anterior synechiae based on GEE models for all variables. [file 40662_2020_212_MOESM2_ESM.docx]

| **Supplementary Table 2. Estimated mean differences in postoperative IOP in nanophthalmic eyes without peripheral anterior synechiae based on GEE models for all variables.** | | | | |
| --- | --- | --- | --- | --- |
| **Variable** | **Univariate GEE models** | | **Multivariate GEE models** | |
|  | **Beta** ^a^ | ***P* value** | **Beta** ^a^ | ***P* value** |
| Age | -0.069 ± 0.032 | 0.032^b^ | -0.046 ± 0.017 | 0.006^b^ |
| Gender | NA | 0.371 |  |  |
| Eye laterality | NA | 0.127 |  |  |
| CCT | -0.001 ± 0.002 | 0.679 |  |  |
| AL | -4.730 ± 2.505 | 0.059 | -1.366 ± 0.114 | <0.001^b^ |
| ACD | -2.464 ± 2.595 | 0.342 |  |  |
| LT | -1.103 ± 1.213 | 0.363 |  |  |
| Preoperative IOP | 0.515 ± 0.226 | 0.023^b^ | 0.280 ± 0.048 | <0.001^b^ |
| Glaucoma surgery | NA | 0.858 |  |  |
| Boomerang-shaped iris | NA | 0.693 |  |  |
| Iris crypt grading | -1.001± 0.707 | 0.157 | -3.290 ± 0.585 | <0.001^b^ |
| SC diameter | -0.055 ± 0.039 | 0.165 | 0.067± 0.001 | <0.001^b^ |
| SC area | -0.001 ± 0.001 | 0.629 |  |  |
| TM thickness | -0.046 ± 0.005 | <0.001^b^ |  |  |
| TM width | -0.001 ± 0.003 | 0.767 |  |  |
| TM area | 0.0001 ± 0.0001 | 0.090 | -0.0002 ± 0.00004 | <0.001^b^ |
| IOP = intraocular pressure; GEE = generalized estimating equation; NA = not applicable; CCT = central corneal thickness; AL= axial length; ACD = anterior chamber depth; LT = lens thickness; SC = Schlemm's canal; TM = trabecular meshwork.  ^a^ Data represent the mean changes ± standard error in postoperative IOP anticipated for each factor. GEE analysis was used.  ^b^ Statistically significant (*P* < 0.05). | | | | |
